# A Role for CARM1-Mediated Histone H3 Arginine Methylation in Protecting Histone Acetylation by Releasing Corepressors from Chromatin

**DOI:** 10.1371/journal.pone.0034692

**Published:** 2012-06-18

**Authors:** Jing Wu, Nan Cui, Rui Wang, Jiwen Li, Jiemin Wong

**Affiliations:** Shanghai Key Laboratory of Regulatory Biology, Institute of Biomedical Sciences and School of Life Sciences, East China Normal University, Shanghai, China; Indiana University School of Medicine, United States of America

## Abstract

Arginine methylation broadly occurs in histones and has been linked to transcriptional regulation, cell cycle regulation and DNA repair. While numerous proteins (histone code effectors) that specifically recognize or read the methylated lysine residues in core histones have been identified, little is known for effectors specific for methylated arginines in histones. In this study, we attempted to identify effector(s) recognizing asymmetrically methylated R17 and R26 in H3, which are catalyzed by CARM1/PRMT4, through an unbiased biochemical approach. Although we have yet to identify such effector using this approach, we find that these modifications function cooperatively with histone acetylation to inhibit the binding of the nucleosome remodeling and deacetylase complex (NuRD) and TIF1 family corepressors to H3 tail in vitro. In support of this finding, we show that overexpression of CARM1 in 293 T cells leads to reduced association of NuRD with chromatin, whereas knockdown of CARM1 in HeLa cells leads to increased association of NuRD with chromatin and decreased level of histone acetylation. Furthermore, in the Carm1−/− MEF cells there is an increased association of NuRD and TIF1β with chromatin and a global decrease in histone acetylation. By chromatin immunoprecipitation assay, we show that overexpression of CARM1 results in reduced association of NuRD complex and TIF1β with an episomal reporter and that CARM1 is required in MEF cells for LPS-induced dissociation of NuRD from a NF-κb target gene. Taking together, our study provides evidence for a role of CARM1-mediated arginine methylation in regulation of histone acetylation and transcription: facilitating transcription by discharging corepressors from chromatin.

## Introduction

Accumulating evidence indicates that histone methylation plays important regulatory roles in all DNA templated biological processes including transcription, DNA replication and repair [1]–[3]. Multiple lysine and arginine residues in core histones can be methylated. Given their chemical nature, lysine residues can be mono-, di- and tri-methylated, whereas arginine can be mono-, asymmetrically and symmetrically di-methylated (me2a and me2s). The availability of multiple lysine and arginine residues for methylation, in combination with different degrees of methylation, endows histone methylation to encode wealthy information and underscores the complexity of methylation regulation [1], [4].

As methyl group is relatively small in size and does not neutralize positive charge in lysine and arginine, methylation is believed to exert regulatory functions in chromatin primarily as docking sites for effectors that recognize and engage physiological functions of the specific methylation [5]–[7]. Since the initial identification of bromodomain-containing proteins as acetylated lysine and HP1 proteins as methylated H3K9 binding proteins [8]–[10], an increasingly large number of effectors have been reported for various methylated lysine residues in core histones [5], [11]–[13]. However, despite broad existence of arginine methylation in histones, upon to now TDRD3 and Dnmt3a are the only proteins reported to bind methylated arginines in histones [14], [15]. TDRD3 was shown to bind both H3R17me2a and H4R3me2a peptides after screening a large panel of known and potential modified histone binding structural motifs [15]. This result suggests that the effectors for methylated arginines may either use different binding motif(s) or are less abundant than effectors for methylated lysines. On the other hand, H3R2 methylation has been shown to impede the binding of effectors to methylated H3K4 [16], raising the possibility that arginine methylation may impede rather than attract binding of effector proteins.

CARM1/PRMT4 functions as a transcriptional coactivator for various transcriptional factors including nuclear receptors and NF-κb [3], [17]–[19]. CARM1 is a type I arginine methyltransferase that catalyzes mono- and asymmetrical dimethylation on R17 and R26 sites in histone H3 and non-histone proteins including CBP/p300, SRC3 and RNA pol II [20]–[24]. Methylation of these non-histone proteins has been linked to, and thus may in part account for, CARM1 coactivator function. Nevertheless, an elegant in vitro study using reconstituted chromatin substrates demonstrated that the ability for CARM1 to stimulate transcriptional activation by p53 depends on arginine methylation on histone H3 [25]. In the same study, CARM1 was shown to stimulate transcription in a step subsequent to p300-mediated histone acetylation. This sequential working model is consistent with a previous study showing that CBP-catalyzed histone acetylation in the PS2 promoter occurs prior to CARM1-mediated H3R17 methylation during estrodial induced activation of PS2 gene [26]. These studies together indicate that CARM1-mediated histone methylation facilitates transcription in the context of histone acetylation. However, how methylation of H3R17 and H3R26 facilitates transcriptional activation in the context of histone acetylation is not known.

In this study, we provide evidence that CARM1-mediated H3R17me2a and H3R26me2a function cooperatively with histone acetylation to inhibit the binding of corepressors and protect chromatin from deacetylation. Thus, our study reveals a novel function for CARM1-mediated arginine methylation: facilitating transcription by discharging corepressors from chromatin.

## Results

### Asymmetrical Dimethylation on Either R17 or Both R17 and R26 does not Appear to Significantly Influence the Binding of Nuclear Proteins to H3 Tail

**Figure 1:**
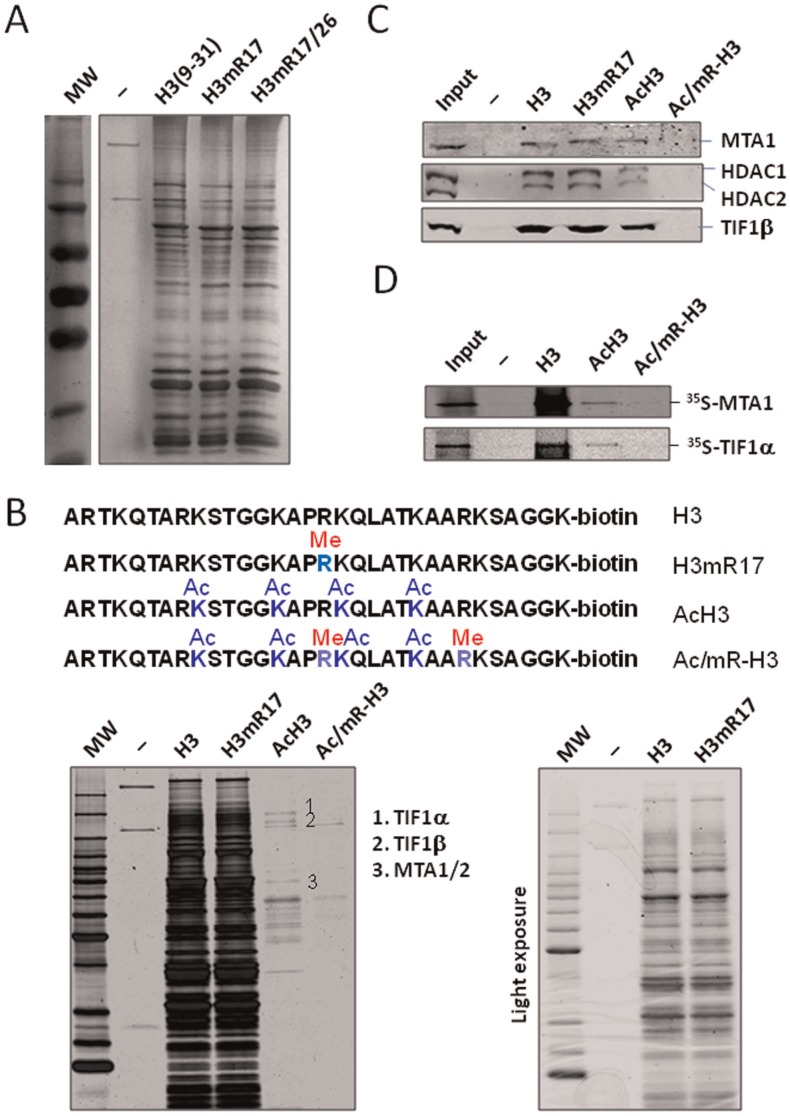
H3R17me2a and H3R26me2a inhibit binding of NuRD and TIF1 family proteins to acetylated H3 tail peptide. (A) Purification of HeLa nuclear proteins using immobilized control and H3 peptides containing either R17me2a (H3mR17) or R17me2a and R26me2a double methylation (H3mR17/26). All peptides correspond to H3 N-terminal tail amino acids 9–29 and have three additional amino acids GGK plus a C-terminal Biotin. The peptides were first immobilized to streptavidin agarose beads and then incubated with HeLa nuclear extracts. The proteins retained by the control beads (−) or immobilized peptides were resolved by 10% SDS-PAGE and visualized by silver staining. (B) Purification of HeLa nuclear proteins using H3 peptides containing either acetylation (AcH3) or both acetylation and R17/R26me2a (Ac/mR-H3). All peptides correspond to H3 tail amino acids 1–29. The bound proteins were resolved by 10% SDS-PAGE and revealed by silver staining. The major bands that were present in the lane of AcH3 but absent in the lane of Ac/mR-H3 were excised and identified by mass spectrometry analysis. The light exposure was shown for proteins bound to H3 and H3 mR17 peptide. Note R17me2a alone did not appear to affect the binding of numerous proteins to H3 peptide. (C) Western blot analysis confirmed the inhibitory effect of H3R17/26me2a on the binding of NuRD complex and TIF1β to acetylated H3 peptide. The proteins bound to various immobilized peptides were analyzed by western blotting using antibodies as indicated. (D) In vitro synthesized MTA1 and TIF1α bound poorly to Ac/mR-H3. MTA1 and TIF1α were synthesized and radiolabled with ^35^S-methionine using transcription and translation coupled reaction (Promega) and subjected to pulldown assay using peptides as indicated. The binding of MTA1 and TIF1α was revealed by autoradiography.

To investigate how the CARM1-catalyzed H3R17me2a and H3R26me2a stimulate transcription in chromatin, we attempted to isolate and identify potential effector(s) that binds specifically H3R17me2a and/or H3R26me2a. We first synthesized biotinylated peptides corresponding to the amino acid sequence 9–31 of H3 N-terminal tail containing either unmodified [H3(9–31)], R17me2a [H3mR17 peptide] or R17me2a + R26me2a [H3mR17/26] peptides. Approximately 5 µg of each peptide was immobilized to streptavidin agarose beads and incubated with HeLa nuclear extracts under relatively mild condition (see Experimental Procedure). After extensive washing, the proteins that bound to immobilized peptides were eluted, resolved by SDS-PAGE and visualized by silver staining. A representative result in Fig. 1A shows that many proteins were retained by immobilized H3(9–31) peptides but not the control beads. Somewhat disappointingly, we did not observe any protein band that was unique to either H3mR17 or H3mR17/26 peptides. Similar results were obtained in multiple efforts under different incubation and washing conditions including salt concentrations ranging from 100 mM to 350 mM and NP40 from 0.05% to 0.5%. These results were very different from what we and others observed for H3 peptides with K4me2/3 or K9me2/3 [27]–[30]. K4 methylation substantially changes the profile of H3 binding proteins, on one hand impeding the binding of NuRD complex and on the other hand enhancing the binding of many effector proteins including CHD1 [31], [32], TFIID [33], Ing2 [34], [35], nardilysin [36] and PHF8 [37], [38]. We thus concluded that asymmetrical dimethylation on either R17 or both R17 and R26 did not appear to significantly influence the binding of nuclear proteins to the amino acids 9–31 of H3 tail.

### Asymmetrical Dimethylation on Both R17 and R26 Acts Cooperatively with Acetylation to Inhibit the Binding of Corepressors NuRD and TIF1 Family Proteins to H3 Tail

Given that the CARM1-catalyzed H3R17me2a and H3R26me2a are likely to facilitate transcription in a step subsequent to histone acetylation [25], [26], R17/26me2a may work together with acetylation as combinatorial code(s) to specify binding of effector(s). To test this hypothesis, we synthesized four new H3 peptides from amino acid 1 to 31. These peptides contain either unmodified (H3 peptide), R17me2a alone (H3mR17 pepetide), acetylation on four (K9, K14, K18 and K23) (AcH3 peptide) frequently acetylated sites, and acetylation plus R17/R26me2a (Ac/mR-H3 peptide)) (Fig. 1B top panel). These peptides were used to purify binding proteins from HeLa nuclear extracts as above. The results in Fig. 1B (see light exposure on right panel) showed that R17me2a modification alone again did not change the binding profile of HeLa nuclear proteins to H3 peptide. Significantly, while much less proteins bound to acH3 peptide in comparison to the H3 peptide, even less proteins bound to the Ac/mR H3 peptide. We subjected the major protein bands that bound to AcH3 but not to Ac/mR-H3 peptide to mass spectrometry analysis and identified them as TIF1Α, TIF1Β and metastasis-associated protein 1 and 2(MTA1/2), respectively.

TIF1α/TRIM24 and TIF1β/KAP1/TRIM28 are related transcriptional cofactors belonging to the tripartite motif family proteins [39]. TIF1α has been implicated in both transcriptional activation and repression. TIF1β is the corepressor for the Krupple family and other transcription factors and has been shown to interact with HDACs, SETDB1 and HP1 [40], [41]. MTA1 and MTA2 are subunits of the NuRD complexes that contain both ATP-dependent chromatin remodeling activity and histone deacetylase activity [42], [43]. In addition to the MTA family proteins (MTA1, MTA2 and MTA3), the NuRD complex contains either a CHD3 or CHD4 helicase subunit, HDAC1/2, RbAP46/48 and several other proteins. Current studies indicate that NuRD is one of the most abundant HDAC complex in HeLa cells and has a broad transcriptional regulatory function. To substantiate our mass spectrometry results described above, we performed pulldown as above and subjected the peptide pulldown materials to Western blot analysis using various antibodies against the components of NuRD complex and TIF1β. The results in Fig. 1C revealed that mR17me2a alone did not affect the binding of MTA1 and TIF1β to H3 peptide. However, less MTA1 and TIF1βbound to the AcH3 peptide than to the H3 peptide. In agreement with the silver staining data in Fig. 1B, Western blot analysis demonstrated that even less MTA1 and TIF1β bound to the Ac/mRH3 peptide. (Fig. 1C). We also analyzed the binding profile of HDAC1/2 and the results in Fig. 1C showed that HDAC1/2 exhibited the same binding profile as MTA1, in agreement with HDAC1/2 being subunits of the NuRD complex. Using in vitro synthesized, ^35^S-methionine labeled MTA1 and TIF1Α, we further showed that these proteins bound poorly to the AcH3 peptide and even worse to the Ac/mR-H3 peptide (Fig. 1D). As NuRD and TIF1β are well established for their corepressor function, these data reveal that H3R17 and H3R26 methylation may function in conjunction with acetylation to impede binding of corepressors to chromatin.

### Overexpression of CARM1 in 293 T Cells Resulted in Reduced Chromatin Association of NuRD and TIF1β

**Figure 2:**
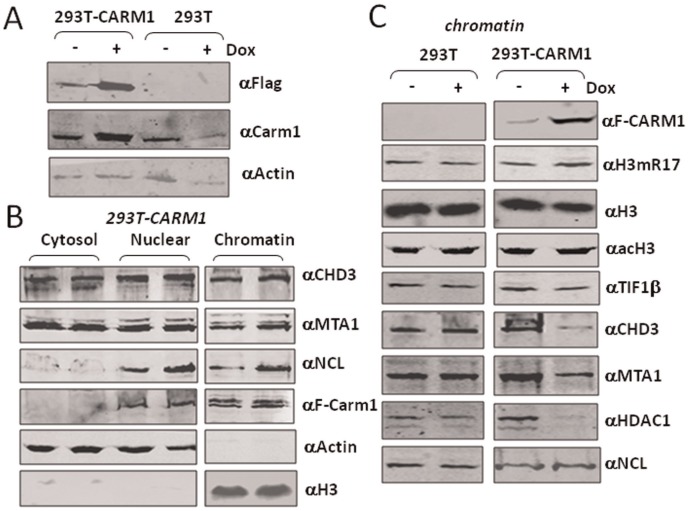
Overexpression of CARM1 in 293 T cells reduced the association of NuRD complex and TIF1β with chromatin. (A) Characterization of Dox inducible 293 T Flip-in CARM1 expression cell line. The 293T-CARM1 and control cells were treated with 0.5 µg/ml Dox for 12 h and the expression of induced expression of Flag-CARM1 was detected by Western blotting using anti-Flag antibody. The levels of total CARM1 were revealed by Western blotting using an anti-CARM1 antibody. (B) Establish the conditions to fractionate the 293T-CARM1 cells into cytosol, nuclear and chromatin fractions. The fractionation was performed in duplicate. (C) Induced expression of CARM1 led to reduced chromatin association of NuRD complex and TIF1β. The control and 293T-CARM1 cell lines were treated without or with Dox for 0.5 µg/ml 12 h and chromatin fractions were prepared and analyzed for histone modifications and association of CARM1, components of NuRD complex, TIF1β and nucleolin as indicated.

To test if the effect of H3R17/R26 methylation on binding of corepressor proteins is physiological relevant, we first tested if overexpression of CARM1 in cells would lead to reduced chromatin association of NuRD complex. Toward this end, we established a doxycycline (Dox) inducible CARM1 expression 293 T (293T-CARM1) cell line using 293 T Flp-In T-Rex cells. Pilot experiments showed that induction of Flag-CARM expression was detectable 4 h after addition of Dox, and increased gradually and reached maximal levels around 24 h (data not shown). To balance the need for induction of Flag-CARM1 and minimizing the potential secondary effect of Flag-CARM1 overexpression, we choose to treat the cells with Dox for 12 h. As shown in Fig. 2A, addition of Dox for 12 h substantially induced the expression of Flag-CARM1. Western blot analysis using a CARM1-specific antibody showed that upon induction the total level of CARM1 increased about 3–4 fold. We next established conditions to fractionate 293T-CARM1 cells into cytosol, nuclear and chromatin fractions (Fig. 2B). As markers for appropriate cellular fractionation, actin was detected in both cytosol and nuclear fractions, whereas H3 was detected only in chromatin. In addition, the components of NuRD complex CHD3 and MTA1 were present in all three fractions, whereas nucleolin and CARM1 were present in nuclear and chromatin fractions.

With the establishment of conditions for cellular fractionation, we prepared the chromatin fractions form the control 293 T and 293T-CARM1 cell lines treated with or without Dox for 12 h. Subsequent Western blot analysis (Fig. 2C) revealed an increased association of Flag-CARM1 with chromatin from 293T-CARM1 cells, in agreement with Flag-CARM1 being induced by Dox treatment. Dox treatment also led to a small, but consistently detectable, increase of H3R17me2a (∼2 fold) in the 293T-CARM1 but not the control 293 T cells. Due to lack of a good antibody H3R26me2a, however we could not demonstrate directly by Western blot analysis whether induced expression of CARM1 also led to increased level of H3R26me2a. To our satisfaction, we found that Dox treatment led to reduced chromatin association of CHD3, MTA1, HDAC1 and TIF1β in the 293T-CARM1 but not the control 293 T cells (Fig. 2C). As a control, the association of nucleolin with chromatin was not significantly affected. These results provide evidence that increased expression of CARM1 can lead to increased H3R17 methylation (possibly also H3R26 methylation) and reduced association of NuRD complex and TIF1β with chromatin.

### Knockdown of CARM1 in HeLa Cells Resulted in Increased Chromatin Association of NuRD and Reduced Histone H3 and H4 Acetylation

**Figure 3:**
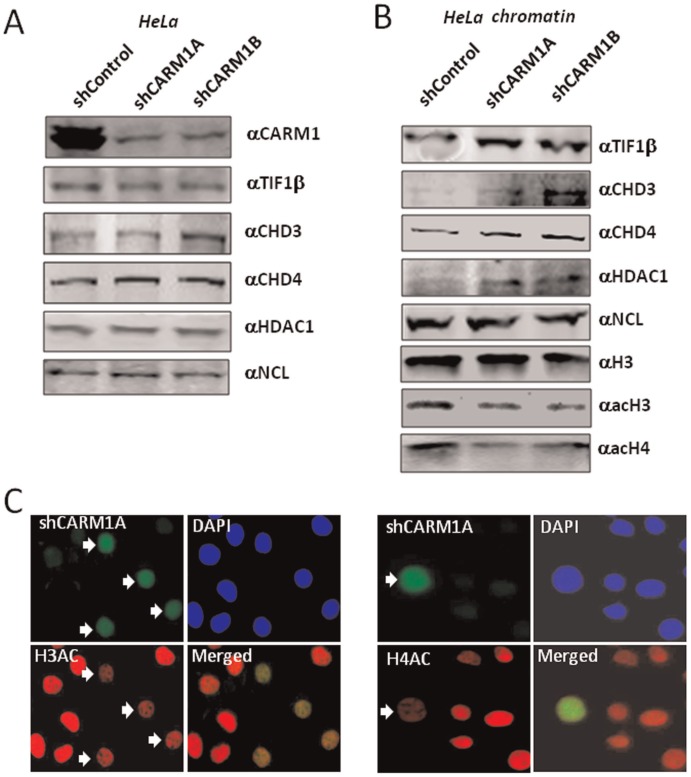
Knockdown of CARM1 in HeLa cells resulted in increased chromatin association of NuRD and TIF1β and reduced histone acetylation. (A) Efficient knockdown of CARM1 in HeLa cells using two shRNAs against CARM1 as revealed by Western blot analysis. The cells were transfected with control or shRNAs against CARM1 for 3 days. The whole cell extracts were prepared and analyzed by Western blotting using antibodies as indicated. Note knockdown of CARM1 did not affect the levels of other proteins tested. (B) Knockdown of CARM1 in HeLa cells resulted in increased chromatin association of NuRD complex and TIF1β and histone hypoacetylation. Chromatin fractions were prepared from HeLa cells treated with control or shRNAs against CARM1 for 3 days and analyzed by Western blotting using antibodies as indicated. (C) Transfection of HeLa cells by shRNA against CARM1 resulted in reduced histone acetylation as revealed by immunofluorescent staining. HeLa cells were transfected with shCARM1A as indicated and subjected to immunofluorescent staining 48 h after transfection. The shRNA transfected cells were GFP positive as the shRNA vector expressed GFP. Note the GFP positive cells were low in acetylated H3 and H4 immunostaining.

To further test the effect of CARM1 on chromatin association of NuRD, we next attempted loss of function experiments by knockdown of CARM1 in HeLa cells using two different shRNAs. HeLa cells were transfected with the control or shCARM1 plasmids and 48 h after transfection the cells were collected and divided into two half, with one for preparation of whole cell extracts and one for preparation of chromatin fractions. Subsequent Western blot analysis of the resulting whole cell extracts revealed that transfection of two different shCARM1 plasmids, but not the control shRNA (against GFP), efficiently reduced the levels of CARM1 in HeLa cells (Fig. 3A). Western blot analysis also revealed that knockdown of CARM1 in HeLa cells did not affect the protein levels of TIF1β, CHD3, CHD4, HDAC1 and nucleolin (Fig. 3A). However, Western blot analysis of the chromatin fractions revealed that knockdown of CARM1 resulted in an increased association of TIF1β, CHD3, CHD4 and HDAC1 with chromatin (Fig. 3B). As a control, the association of nucleolin with chromatin was not changed (Fig. 3B).

As NuRD is one of the most abundant histone deacetylase complexes in HeLa cells, we tested whether increased chromatin association of NuRD complex upon knockdown of CARM1 would affect histone acetylation in chromatin. Significantly, we found that knockdown of CARM1 indeed resulted in decreased levels of acetylated H3 and H4 (Fig. 3B). The effect of knockdown of CARM1 on histone acetylation was further confirmed by immunofluorecent staining using antibodies against acetylated H3 and H4 (Fig. 3C). Together these results indicate that knockdown of CARM1 in HeLa cells results in increased association of NuRD and TIF1β with chromatin and histone hypoacetylation.

### Chromatin from the *Carm1−/−* MEF Cells Shows Increased Corepressor Association and Histone Hypoacetylation

**Figure 4:**
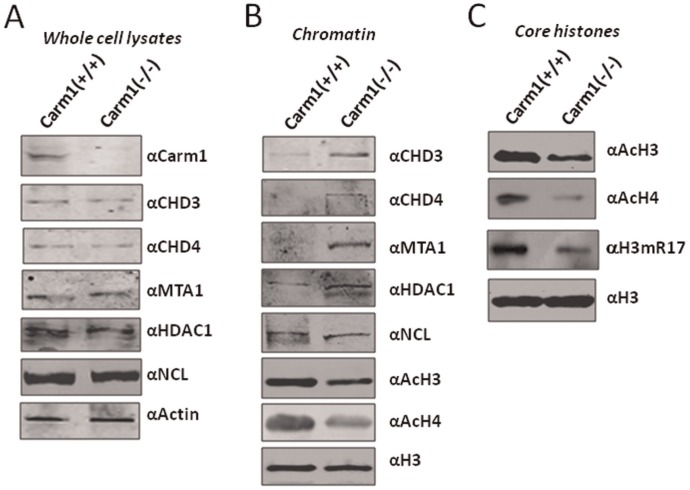
Increased chromatin association of NuRD and TIF1β and decreased histone acetylation in *Carm1* knockout MEF cells. (A) Whole cell extracts were prepared from Carm1+/+ and Carm1−/− MEFs and subjected to Western blot analysis using antibodies as indicated. (B) Chromatin fractions were prepared from Carm1+/+ and Carm1−/− MEFs and analyzed by Western blotting using antibodies as indicated. (C) Western blot analysis of core histones prepared from the wild-type and Carm1 knockout MEFs by acid extraction.

To further substantiate our finding that CARM1 regulates the association of NuRD complex with chromatin and consequently histone acetylation, we next made use of paired wild-type and *Carm1−/−* MEF cells generated by Mark Bedford’s group [21]. We prepared the whole cell extracts and chromatin fractions from these cell lines. First, we confirmed by Western blot analysis the absence of CARM1 proteins in the whole cell extracts derived from the *Carm1−/−* MEF cells (Fig. 4A). No obvious differences in the levels of total proteins were observed for CHD3, CHD4, MTA1, HDAC1 and nucleolin between the wild-type and *Carm1−/−* MEFs. However, Western blot analysis of the chromatin fractions revealed increased association of CHD3, CHD4, MTA1 and HDAC1 with chromatin from the *Carm1−/−* MEF cells (Fig. 4B). As a control for specificity, no increased association was observed for nucleolin. In agreement with the shCARM1 results from HeLa cells, the chromatin prepared from the *Carm1−/−* cells was found to contain hypoacetylated histones H3 and H4 in comparison to the chromatin from the wild-type MEF cells (Fig. 4B). This hypoacetylation phenotype was further confirmed by analysis of the core histones prepared from both wild-type and Carm1−/− MEF cells using acid extraction method (Fig. 4C). Note that Western blot analysis confirmed reduced level of H3R17me2a in the core histones derived from the *Carm1−/−* cells (Fig. 4C).

### Expression of CARM1 Dissociates NuRD and TIF1β from Chromatin

**Figure 5:**
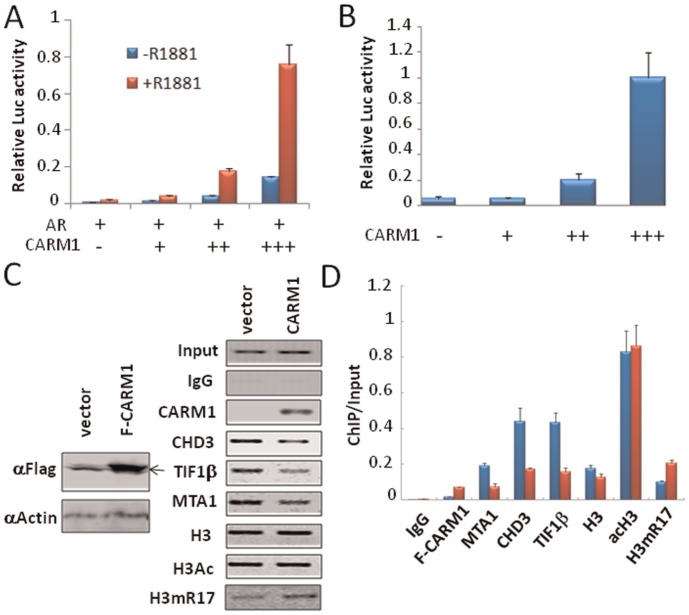
Chromatin immunoprecipitation (ChIP) assay demonstrated that expression of CARM1 resulted in reduced chromatin association of NuRD and TIF1β. (A) Expression of CARM1 facilitates transcriptional activation by AR. HeLa cells were transfected with 100 ng pREP7-MMTV-Luc reporter, 20 ng pSG5-Flag-AR plus 0, 100 ng, 200 ng or 400 ng of pSG5-CARM1 and treated with or without 10 nM R1881 overnight. The relative luciferase activities were based on triplicates of two experiments. (B) Expression of CARM1 alone activates transcription from pREP7-MMTV-Luc reporter. pREP7-MMTV-Luc reporter was cotransfected with an increasing amount of pSG5-Flag-CARM1 plasmid. The relative luciferase activities from two independent experiments were shown. (C) ChIP analysis revealed that expression of CARM1 resulted in reduced association of NuRD and TIF1β with chromatin. HeLa cells were transfected with pREP7-MMTV-Luc reporter alone or plus 300 ng pSG5-Flag-CARM1. One day after transfection, the cells were collected and proceeded for ChIP analysis using antibodies as indicated. Left panel shows Western blot data for expression of Flag-CARM1. Arrow marks a non-specific band. PCR reactions were performed using a pair of MMTV-LTR specific primers and the products were revealed by agarose gel electrophoresis. (D) ChIP products in C) were analyzed by quantitative PCR.

The above results reveal that overexpression and knockdown or knockout of CARM1 all grossly affect the association of NuRD and TIF1β with chromatin and consequently histone acetylation. We next attempted to test the effect of CARM1 on corepressor chromatin association using a modified episomal reporter by chromatin immunoprecipitation (ChIP) assay. This pREP7 based episomal reporter (pREP7-MMTV-Luc) can replicate and maintain as minichromosomes in cells, because the vector contains an Epstein-Barr virus replication origin (oriP) and EBNA gene required for oriP based replication. As CARM1 is a coactivator for androgen receptor (AR) [17], the initial thought was to test if expression of CARM1 facilitates the dissociation of corepressors NuRD and TIF1β from the reporter in the presence of AR. The results in Fig. 5A showed that expression of CARM1 enhanced, as expected, the hormone-dependent (+R1881) transcriptional activation from the pREP7-MMTV-Luc reporter by AR. However, we also noticed that expression of CARM1 in the absence of R1881 increased the levels of luciferase activity, suggesting that expression of CARM1 alone may moderately activate transcription. Indeed, we found that expression of CARM1 alone enhanced the luciferase activity from the reporter in a dose-dependent manner (Fig. 5B). This observation allowed us to test the effect of overexpression of CARM1 alone on the association of corepressors NuRD and TIF1β with chromatin, thus avoiding the complication resulting from the action of various coactivators recruited by AR. We therefore expressed the reporter with and without CARM1 and carried out chromatin immunoprecipitation assay to assess the effect CARM1 expression on the association of NuRD and TIF1β with chromatin. The representative semi-quantitative PCR results in Fig. 5C showed that expression of CARM1 led to reduced association of CHD3, MTA1 and TIF1β and increased association of CARM1 and increased level of H3R17me2. The above results were further verified by quantitative PCR analysis as shown in Fig. 5D.

**Figure 6:**
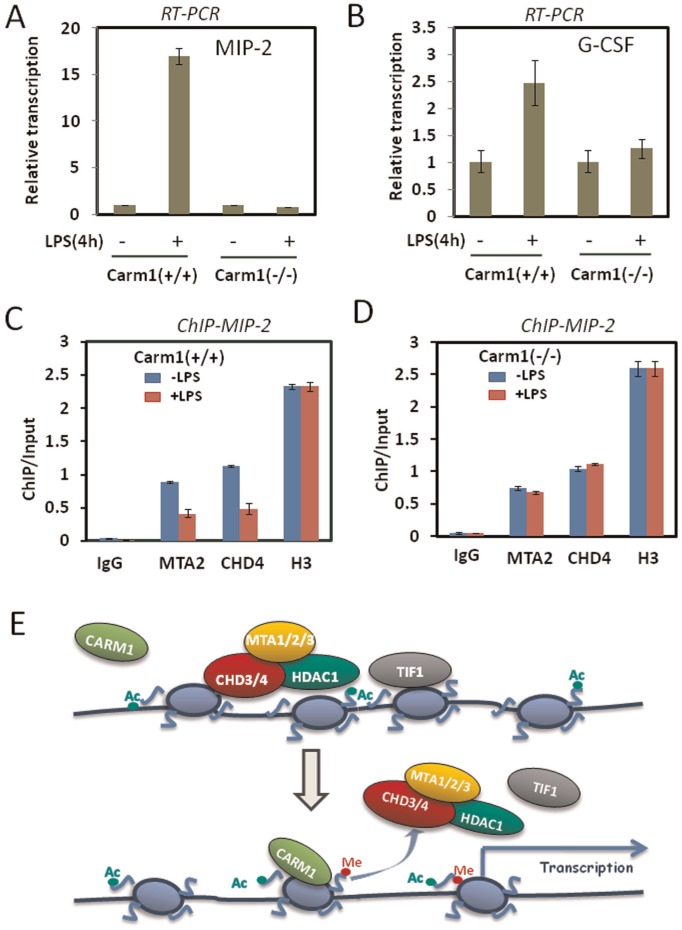
CARM1 regulates the chromatin association of NuRD with a NF-κb-dependent gene. (A) and (B) The Carm1(+/+) and Carm1(−/−) MEF cells were treated with or without 10 µg/ml LPS for 4 h. The total RNAs were prepared and analyzed by quantitative RT-PCR for LPS induction of NF-κb-regulated target genes MIP2 and GCSF respectively. (C) and (D) The ChIP analyses were performed for the MIP2 promoter using Carm1(+/+) and Carm1(−/−) MEF cells treated with or without LPS for 4 h. Note that the association of NuRD complex was determined using antibodies against MTA2 and CHD4 antibodies, as the antibodies against MTA1 and CHD3 used in Fig. 5 did not work well for ChIP with MEF cells. The percentage of DNA immunoprecipitated with each antibody was calculated relative to the ChIP input DNA. Values are means±SD (standard deviation) derived from three independent ChIP experiments (E) A working model illustrating how CARM1 facilitates dissociation of NuRD and TIF1β from chromatin. CARM1 catalyzes methylation on H3R17 and H3R26. These modifications in the context of acetylation discharge NuRD and TIF1β from chromatin, which in turn protects histone acetylation and enhances transcriptional activation.

To test further a role for CARM1 in regulating the association of NuRD corepressor complex with chromatin in a physiological relevant condition, we next made use of Carm1+/+ and Carm1−/− MEF cells. A previous study has identified a subset of NF-κb target genes including MIP-2 and G-CSF whose expression is induced by LPS in the Carm1+/+ but not in Carm1−/− MEF cells [19]. These MEF cells were treated with or without 10 µg/ml lipopolysaccharide (LPS) for 4 h and then collected for preparation of total RNAs. Quantitative RT-PCR analysis confirmed that LPS-induced MIP-2 expression was severely impaired (Fig. 6A), whereas the expression of another NF-κb target gene G-CSF was less affected (Fig. 6B). Subsequent ChIP analysis was then focused on the promoter of MIP-2 gene and the results in Fig. 6C show that LPS treatment led to reduced association of MTA2 and CHD4 with chromatin. However, ChIP analysis revealed that LPS treatment did not result in reduced association of MTA2 and CHD4 with chromatin (Fig. 6D). These results provide further evidence that CARM1 regulates the chromatin association of NuRD complex.

## Discussion

Arginine methylation broadly occurs in histones and non-histone proteins [3]. Lysine methylation on various sites of histones plays diverse functions that have been linked to its ability to recruit distinct binding or effector proteins. Indeed, numerous proteins with chromo, PHD, WD40 or tudor domain have been identified collectively as effectors for methylated H3K4, H3K9, H3K27, H3K36 and H4K20 [5]–[7]. In contrast, TDRD3 is currently the lonely effector identified for arginine-methylated histones from a protein microarray [16]. This result suggests that the effectors for methylated arginines may either use different binding motif(s) or are less abundant than effectors for methylated lysines. In this study, we employed an unbiased affinity purification approach to identify from HeLa nuclear extracts the effectors for H3R17me2a and H3R26me2a. This same approach has led us to the identification of large numbers of H3K4me2- and H3K9me2-binding proteins [30]. However, despite of our extensive efforts, we have yet to identify a protein that binds specifically or preferentially to H3R17me2a and/or H3R26me2a. Careful comparison of protein band profiles of sliver staining gels did not reveal any obvious protein band that was enriched by H3R17me2a and/or H3R26me2a peptides but not by the control unmodified peptide. This observation suggests that H3R17me2a and/or H3R26me2a may not exert effect in chromatin by serving as docking sites for major chromatin associated proteins. A caveat in our experiment is that we could not exclude the possibility that the effectors for H3R17me2a and H3R26me2a (such as TDRD3) might present in low abundance and thus were masked in gels by other more abundant H3 peptide binding proteins.

Given the previous observation that H3R17me2a and H3R26me2a is likely to function in conjunction with histone acetylation [25], [26], we therefore attempted to identify effector(s) for these combinatorial codes. We instead find that H3R17me2a and H3R26me2a, in conjunction with K9, K14, K18 and K23 acetylation, substantially reduced the binding of corepressors NuRD and TIF1α and TIF1β to H3 tail (Fig. 1C). Although we did not attempt to determine the protein identities for the additional bands that bound to the acH3 but not ac/mRH3 peptide by mass spectrometry, Western blot analysis suggested that they were primarily the components of NuRD complex including HDAC1 and HDAC2. Based on our results and the previous report that H3R2me2 inhibits the binding of several H3K4me2 effectors to H3 tail [16], we propose that arginine methylation in histones is perhaps more likely to inhibit rather than recruit effector proteins. Mechanistically, because arginine contains five potential hydrogen bond donors that can form favorable interaction with biological hydrogen bond acceptors, arginine residues in histones are likely to widely engage in interaction with histone binding proteins. Methylation on arginine not only changes its shape but also affects the number of available hydrogen bond donors, which in turn may inhibit the arginine-engaged protein-protein interaction. Perhaps only for a small group of tudor domain proteins including TDRD3, arginine methylation facilitates protein-protein interaction through hydrophobic interaction between methyl group and aromatic rings in tudor domain.

As NuRD and TIF1β are transcriptional corepressors broadly involved in transcriptional repression [40]–[43], our finding immediately provides a potential mechanism for transcriptional activation mediated by CARM1-catalyzed histone arginine methylation: dissociating corepressors NuRD and TIF1β from chromatin. We therefore focused our effort on testing whether CARM1 indeed regulates the association of NuRD and TIF1β with chromatin in cells. Several lines of evidence support that CARM1 regulates the association of NuRD and TIF1β with chromatin in cells. First, we show that induced overexpression of CARM1 in 293 T cells led to reduced association of NuRD with chromatin (Fig. 2C). Second, knockdown of CARM1 in HeLa cells resulted in increased association of NuRD and TIF1β with chromatin (Fig. 3B). Third, we observed in Carm1−/− MEF cells an increased association of NuRD and TIF1β with chromatin (Fig. 4B and 4C). Fourth, by ChIP analysis of a chromatinized episomal reporter (Fig. 5C and 5D) and an endogenous NF-κb target gene (Fig. 6C and 6D), we show that CARM1 regulates the association of NuRD with chromatin. Together these in vitro and in vivo results provide compelling evidence that CARM1 regulates the association of NuRD and possibly other corepressor proteins such as TIF1β with chromatin.

Another important observation in this study is that knockdown of CARM1 in HeLa cells and knockout of CARM1 in MEF cells lead to histone hypoacetylation. To our knowledge, this is the first report that CARM1 has a role in regulating global histone acetylation. This finding is consistent with a role of CARM1 in regulating chromatin association of the chromatin remodeling and histone deacetylase complex NuRD and TIF1β in these cells, although we cannot rule out the possibility that CARM1 may also regulate histone acetylation by influencing the HAT activities such as CBP/p300. It is noteworthy that overexpression of CARM1 did not appear to affect histone acetylation in 293 T cells (Fig. 2C), although it did lead to reduced association of NuRD with chromatin. Similarly, overexpression of CARM1 did not result in histone hyperacetylation in episomal reporter (Fig. 5C and 5D). Thus, whereas increased chromatin association of NuRD complex correlates a reduced histone acetylation, reduced chromatin association of NuRD does not necessarily lead to a global increase in histone acetylation, suggesting that the global levels of histone acetylation are likely regulated by additional factors such as the activity of other HDACs and/or limited activities of histone acetyltransferases.

It is also noteworthy we observe in this study that acetylation on K9, K14, K18 and K23 drastically reduces the binding of HeLa nuclear proteins to H3 tail. This result is consistent with previous reports [44] and suggests that many proteins bind to H3 tail peptide through hydrophilic interactions with positive charged lysine residues. Although a few proteins bound to the acH3 peptide as shown in Fig. 1B, these proteins does not appear to bind specifically to acH3 peptide, because among them are TIF1α, TIF1β and MTA1, which clearly bound more avidly to H3 peptide than to acH3 peptide (Fig. 1C and 1D).

Taking together, both our in vitro and in vivo data support a working model in Fig. 6E. In this model, CARM1-mediated H3R17me2a and H3R26me2a has a role in releasing corepressors from chromatin in conjunction with histone acetylation. This model is consistent with previous studies showing that CARM1 and its catalyzed H3R17/26 methylation facilitate transcriptional activation subsequent to the step of histone acetylation [23], [24]. Our study therefore adds a new flavor to the mechanisms by which CARM1 regulates transcription: relief of repression by NuRD and TIF1 family proteins. Our study also uncovers a role of CARM1 in protecting acetylated histones from deacetylation by NuRD complex.

## Materials and Methods

### Plasmids, Antibody, Cell Lines, Transfection and Stable Cell Line

The CARM1 expression plasmids pCMV-HA-CARM1 and pcDNA5/FRT/TO-CARM1 were constructed by cloning the full-length mouse CARM1 into pCMV-HA and pcDNA5/FRT/TO vector. The ppyCAGIP-AR was constructed by inserting the full-length AR amplified from pSG5-AR into the vector ppyCAGIP. The pREP7-MMTV-luc episomal reporter was constructed by replacing the original RSV-LTR with MMTV-LTR-Luc from pMMTV-LTR-Luc reporter. All constructs were verified by DNA sequencing.

The commercial antibodies used were H3, acH3, acH4, H3R17me2a and TIF1β/KAP1 from Abcam (Cambridge, MA, USA); HA from Roche Molecular Biochemicals; nucleolin and MTA1 from eBioscience (San Diego, CA) and mouse monoclonal CARM1 antibody from AbMart (Shanghai, China). Rabbit polyclonal anti-HDAC1 antibody was a kind gift from Dr Jiangou Song (Shanghai Institute of Biochemistry and Cell Biology, Shanghai, China). Antibodies against CHD3 and CHD4 were kindly provided by Dr. Weidong Wang (National Institute of Aging, Baltimore, USA). Antibody against MTA2 was kindly provided by Dr. Ping Zhu (The Institute of Biophysics, Chinese Academy of Sciences, Beijing). NPM1 antibody was generated by immunizing rabbits with GST-NPM1.

HeLa and 293 T cell lines were obtained from the American Type Culture Collection (ATCC, Manassas, VA). The 293 T Flp-In T-Rex cell line was obtained from Invitrogen Corporation (Carlsbad, CA) and the CARM1(+/+) and CARM1(−/−) MEF cell lines were kindly provided by Dr. Mark Bedford (MD Anderson Cancer Center, Smithville, TX). HeLa, 293 T, 293 T Flp-In T-Rex, CARM1(+/+) and CARM1(−/−) MEF cell lines were maintained routinely in Dulbecco’s modified Eagle’s medium (DMEM) supplemented with 10% fetal bovine serum. Transient transfections in 293 T and HeLa cells were performed using lipofectamine 2000 (Invitrogen, Carlsbad, CA) according to the manufacturer’s instructions.

### Isolation of Effectors from HeLa Nuclear Extracts Using Biotinylated H3 Peptides, Mass Spectrometry and Western Blot Analysis

Nuclear extracts were prepared from HeLa cells by the protocol of Dignam et al [45]. Biotinylated histone tail peptides containing methylated arginines or acetylated lysines were synthesized and purified by Beijing Scilight Biotechnology Ltd. Co. Purification of corresponding H3 peptide binding proteins from HeLa nuclear extracts and mass spectrometry were carried out essentially as described [29]. Western blot analysis was performed using various antibodies as described [46].

### Pull-down Assay with in Vitro Synthesized Proteins

Radiolabeled proteins were generated with the TNT coupled reticulocyte lysate system (Promega). *In vitro* pull-down assays were carried out by incubating the *in vitro* translated products with different modified histone H3 peptides at 4°C for 2 h. After extensive washing, the products were resolved by 10% SDS–PAGE and the presence of proteins was revealed by autoradiography.

### Generation of the Flag-CARM1 Inducible Stable Cell Line and Induction Flag-CARM1 Expression

An inducible stable Flag-CARM1 cell line was generated by transfecting the 293 T Flp-In T-Rex cells with pcDNA5/FRT/TO-Flag-CARM1 and pOG44 according to the manufacturer’s instruction from Invitrogen. To induce expression of Flag-CARM1 in this cell line, doxycycline was added to the medium at a final concentration of 1 µg/ml. The cells were cultured for additional 4–24 hours before harvested and analyzed for Flag-CARM1 expression and cellular fractionation.

### Preparation of Cytosol, Nuclear Extract and Chromatin

To fractionate cellular contents into cytosol, nuclear and chromatin fractions, cultured cells were collected by centrifugation and washed twice with ice-cold PBS. The pellets were resuspended in 2 packed cell volume of solution A (20 mM Tris, pH 8.0, 150 mM NaCl, 1% NP-40, 1 mM DTT and protease inhibitors), incubated on ice for 10 minutes and centrifuged at 4000 rpm for 5 min at 4°C. The supernatants were collected and designated as cytosol fractions. The pellets were washed with solution A once and the resuspended in 2 packed cell volume of solution B (20 mM Tris, pH 8.0, 450 mM NaCl, 1% NP-40, 1 mM DTT and protease inhibitors). After incubation on ice for 10 minutes, the samples were centrifuged at 12000 rpm for 10 min at 4°C. The resulting supernatants were collected and designated as nuclear extracts. The pellets were washed once with solution A, resuspended in 2 volume of 1× SDS loading buffer and designated as chromatin fractions. The samples were boiled before electrophoresis.

### Acid Extraction of Histones

The preparation of core histones by acid extraction was performed as described [47]. The histone preparations were resolved by 18% SDS-PAGE followed by Western blot analysis.

### Luciferase Reporter Assays and RT-PCR Analysis

Transfections for luciferase activity assay were performed in 24-well plates using lipofectamine 2000 (Invitrogen). 48 hours after transcfection, luciferase activity in the cell lysates was determined by using a commercial luciferase reporter assay system (Promega, WI, USA). In experiments with AR, R1881 was added at a final concentration of 10 nM 8 h before cells were harvested for luciferase activity assay. The luciferase activity was normalized against the protein concentration. For analysis of LPS-induced transcriptional activation of MIP-2 and G-CSF genes in Carm1(+/+) and Carm1(−/−) MEF cells, the cells were treated with 10 µg/ml LPS (Sigma) for 4 h before cells were collected for quantitative RT-PCR analysis. The primers are as follow: MIP-2 forward CCGCCCAGACAGAAGTCATAGC, MIP-2 reverse CAGACAGCGAGGCACATCAGG, G-CSF forward GCAGGCTCTATCGGGTATTTCC and G-CSF reverse GCTGGAAGGCAGAAGTGAAGG.

### Chromatin Immunoprecipitation Assay

For chromatin immunoprecipitation (ChIP) assay with 293 T cells transiently transfected with the pREP7-MMTV-luciferase reporter plus pCMV-HA-CARM1 or its vector control, the cells were treated with 1% formaldehyde 48 h after transfection for 10 minutes at RT. The cells were then harvested and ChIP assays were performed essentially as described [48]. The ChIP primer sequences for MMTV-LTR are: TTTCCCCAAAAGTGCATTCCT and CCCTCCCTTTTCGTGAAAGACT. For ChIP assay using Carm1(+/+) and Carm1(−/−) MEF cells, the MEF cells were treated with 10 µg/ml LPS (Sigma) for 4 h before cells were collected for ChIP analysis as above. The ChIP primers for the MIP-2 promoter are GACCACCAAGTCTCTGTTTCTTGA and TGTCATATCCTTCTACCCGACTTG.
